# What the future holds: cystic fibrosis and aging

**DOI:** 10.3389/fmed.2023.1340388

**Published:** 2024-01-08

**Authors:** Sydney Blankenship, Aaron R. Landis, Emily Harrison Williams, Jacelyn E. Peabody Lever, Bryan Garcia, George Solomon, Stefanie Krick

**Affiliations:** ^1^Division of Pulmonary, Allergy, and Critical Care Medicine, Department of Medicine, The University of Alabama at Birmingham, Birmingham, AL, United States; ^2^Gregory Fleming James Cystic Fibrosis Research Center, The University of Alabama at Birmingham, Birmingham, AL, United States

**Keywords:** aging, cystic fibrosis, chronic inflammation, comorbidities, diabetes, cognitive dysfunction, modulator therapy

## Abstract

Cystic fibrosis (CF) is one of the most common genetic diseases with around 70,000 affected patients worldwide. CF is a multisystem disease caused by a mutation in the CF transmembrane conductance regulator gene, which has led to a significant decrease in life expectancy and a marked impairment in the quality of life for people with CF (pwCF). In recent years, the use of highly effective CFTR modulator therapy (HEMT) has led to improved pulmonary function, fewer CF exacerbations, lower symptom burden, and increased weight. This has coincided with an increased life expectancy for pwCF, with mean age of survival being now in the 50s. This being a major breakthrough, which the CF population has hoped for, pwCF are now facing new challenges by growing old with a chronic respiratory disease. In this mini review, we are attempting to summarize the current knowledge of the aging process and its effect on CF disease and its manifestations including new developments, the current research gaps and potential future developments in the field to allow healthy aging for the CF community.

## Introduction

Cystic Fibrosis (CF) is caused by mutations in the CF transmembrane conductance regulator (CFTR) gene, which encodes an epithelial chloride channel as well as modulates the activity of epithelial sodium channels (ENaC) (1–3). CFTR dysfunction leads to impaired balance of ion and fluid in affected cells of sweat glands, airways, pancreas, and other organs leading to acute and chronic complications associated with mucociliary dysfunction such as respiratory infections, respiratory failure, distal intestinal obstruction syndrome, as well as the development of CF-related glucose intolerance and diabetes mellitus (2, 4, 5). From early on people with CF (pwCF) and their caretakers are followed and treated by a highly interdisciplinary medical team to aid with preservation of lung function and overall health. Respiratory clearance therapies and inhaled antibiotics have led to a significant increase in life expectancy, though the introduction and approval of highly effective CFTR modulator therapy (HEMT), specifically Elexacaftor–Tezacaftor–Ivacaftor (ETI or Trikafta^®^), further improved not only the lung function and exacerbation frequency but also the weight and symptoms in pwCF (6–8). PwCF are now living productive lives in their 50s and with the approval of HEMT for children starting at 2 years, life expectancy will increase even further (9). This being a major breakthrough, which the CF population has hoped for, pwCF are now facing new challenges by growing old with a chronic respiratory disease. Up to this point, there are currently only few guidelines in place that address the aging CF population, including advanced lung disease and cancer screening, but there is little known about the effects of aging and aging related pathways in the care of pwCF. In this review, we will summarize important aging and CF related complications and their current treatment options in addition to pointing out major gaps in knowledge, which will need to be addressed in the future.

## CF- and aging-related comorbidities

Since the world population has been aging, especially in the Developing World, the scientific and medical community have been focusing to better understand underlying cellular aging pathomechanisms. The ultimate goal is to be able to promote healthy aging without the development of aging-associated chronic illnesses to improve global quality of life (10). Respiratory diseases are well known to be associated with aging with chronic obstructive pulmonary disease (COPD) being responsible for over 3 million deaths worldwide and followed by lower respiratory tract infections and lung cancer (11). In addition, both COPD and idiopathic pulmonary fibrosis (IPF) have been recognized as aging related lung diseases, being not only more common in the elderly but also showing activation of molecular aging pathways such as increased oxidative stress, cell senescence, mitochondrial dysfunction and apoptotic resistance (12, 13). Furthermore, cardiovascular disease including ischemic heart disease and stroke are leading causes of death in the elderly with hypertension as one of the risk factors (14). Additional aging related comorbidities or diseases that limit “healthy” aging are bone disease, diabetes and obesity (15, 16). Polypharmacy has also been recognized as a global risk factor for the elderly due to greater risk for adverse drug reactions and the higher numbers of drugs used (17). In the following paragraphs, we focus on common health complications that are emerging in the adolescent and “older” pwCF to discuss current knowledge and potential developments.

## Respiratory disease

Respiratory disease manifestations are the major contributor to an impaired quality and quantity of life for most pwCF (18). An impairment in respiratory function can start early on in life leading to pulmonary CF exacerbations and frequent hospitalizations. Children are often colonized with *Staphylococcus aureus* (19), whereas later in life, colonization with *Pseudomonas aeruginosa* (Psa) increases and chronic Psa infection is associated with accelerated lung function decline and increased mortality (20). In the era of CFTR modulator therapy, lower respiratory tract infections remain a key contributor to CF morbidity and mortality (21). There are currently no long-term studies, but after the introduction of highly effective CFTR modulator therapy (HEMT) consisting of Elexacaftor, Tezacaftor and Ivacaftor (ETI or Trikafta®), lung function and quality of life of pwCF significantly improved and the majority those patients, who showed significant benefits did not report productive cough any more. Pallenberg et al., reported that after 1 year of ETI treatment, shotgun metagenome sequencing of respiratory secretions demonstrated a significantly reduced bacterial load (22). Two recent studies also showed the benefit of ETI by significantly improving mucus properties and pathogenic load and inflammation thereby shifting the “CF sputum proteome” towards healthy, but not improving infections and reaching “healthy” (23, 24). Those studies are very promising but still only over a shorter period of time and this warrants further studies to assess long term effects of ETI treatment. Extrapolating from studies using previous generations of CFTR modulators, Durfey et al. demonstrated that Ivacaftor in combination with an intensive antibiotic course over months could clear chronic Psa and *Staphylococcus aureus* lung infections in subjects, who were highly responsive to Ivacaftor, but this effect was only transient in subjects who remained infected (25). Therefore, the future holds promise, especially with the development of genetic therapies and further advanced CFTR modulators that restoration of CFTR activity can aid in attenuating respiratory infections in CF, but a majority of the adult pwCF will remain infected with different multidrug resistant airway bacteria and will need additional therapies they can tolerate to improve their quality and quantity of life.

## Cardiovascular disease

Cardiovascular disease, especially left heart disease, and associated risk factors such as hypertension have not been prevalent in pwCF since CF resulted in premature mortality during the times of those studies: Fraser et al., conducted a small study with 18 patients in 1999 trying to assess the prevalence of pulmonary hypertension and cardiac dysfunction in adult pwCF and severe lung disease and could show that pwCF and severe stable lung disease had a well maintained left and right ventricular function in the absence of coronary artery disease. The development of pulmonary hypertension is strongly associated with hypoxemia (26).

Many studies did not describe left ventricular dysfunction, though there is some evidence that it might be present during exercise in the pediatric CF population, which seemed to be associated with lung disease severity (27). Interestingly, there are several case reports describing coronary artery disease in pwCF and possible atherosclerosis (28–30). Although pwCF express malabsorption of fat, there is evidence of dysregulation in lipids and in the HEMT era and pwCF living longer, improved weight gain and improved fat absorption, we will need to develop preventative strategies for cardiovascular complications.

## Diabetes

CF-related diabetes (CFRD) is a prevalent CF-associated comorbidity and affects more than half of the pwCF (31). The marked increase in CFRD is thought to be due to the fact that pwCF are now living into adulthood (32). With the median life expectancy now surpassing 50 years, this will be an even more important comorbidity to address in an aging CF population. Unlike type 1 or type 2 diabetes, the pathophysiology of CFRD is not completely understood. It is likely multifactorial related to both β-cell dysfunction and a reduction in islet cell mass and ongoing chronic inflammation (33, 34). Regardless of etiology, it has been repeatedly proven that CFRD increases morbidity and mortality in CF patients (35). Unlike type 1 and type 2 diabetes, poor outcomes are typically due to infections and pulmonary exacerbations as opposed to diabetic ketoacidosis or microvascular disease (35, 36). Additionally, it is important to note that nutritional therapy for CFRD differs from T1DM and T2DM. Because insulin deficiency is the primary driver of the disease, weight loss and carbohydrate restriction has not been shown to improve outcomes or slow progression. Instead, it has been recommended that patients consume a high calorie diet with salt and fat consumption until recently, when HEMT were introduced and pwCF started gaining weight (37). Therefore, the presentation of CFRD will most likely change and could potentially behave like a CFRD/T2DM overlap syndrome in a normal weight or even obese CF population. This will lead to an increased burden in comorbidities in those patients, which will require further characterization to find appropriate and potentially individualized treatments to prolong life and improve the quality of life of pwCF with CFRD when they age.

## Cancer

As the CF population begins to age, there has been emerging evidence regarding the population’s increased incidence of cancer, most significantly of gastrointestinal origin (38); of note, this increased risk is furthermore compounded in the setting of solid organ transplant (SOT) with approximately 6%–8% of pwCF worldwide undergoing lung transplantation (39). In one of the largest observational studies to uncover the association between cancer and CF in the United States, there was a comparable risk of cancer in CF patients and the general population. However, when stratifying risk based on type of cancer, gastrointestinal cancers, including both esophageal and colorectal cancers, were on average nearly 3.5 times more likely to be seen in pwCF (40). CFTR dysfunction in the gastrointestinal tract has also been shown to predispose to chronic inflammatory changes (41) and most recently, ETI therapy has shown to improve not only gastrointestinal symptoms, but also fecal markers of inflammation in the PROMISE-GI study (42). Therefore, it will be interesting to see whether the cancer risk will change in pwCF on ETI therapy.

While obvious incidences of certain cancers are increased with immunosuppression following solid organ transplantation (SOT), it is important to frame clinical relevance of this with absolute risk. For example, the literature involving pwCF’s risk of cancer as it relates to SOT revolves largely around lung transplantation. In a 2016 observational study of lung transplant patients, there was an associated 10% increased risk of all types of cancer in the CF patient group with most significant risk involving colorectal and esophageal cancer (43).

The reason of SOT patients having increased risk of developing cancer is likely multifactorial. First, an intrinsic increased risk exists within pwCF (as discussed previously), likely related to a persistent pro-inflammatory state over time (41). Patients with more clinically severe disease are those who are most likely to undergo transplantation. Second, SOT further increases the risk due to dampening the immune system’s ability to identify and irradicate pre-cancerous cells.

Given the mounting evidence of higher rates of colorectal cancer in pwCF, Gastroenterology published a 2018 consensus recommendation regarding screening. These guidelines included, most significantly, colonoscopy as screening exam of choice with the beginning of screening in non-SOT CF patients at 40 years of age, and to begin screening in SOT CF patients who are 30 years of age and older, 2 years following initial transplantation (44). As the population continues to age and change with time, it will be important to continue monitoring rates of cancer in pwCF in order to adjust guidelines appropriately and potentially increase frequency of screening depending on further clinical studies in this field.

## Polypharmacy and medication adverse effects

Prior to recent years, treatment of CF consisted primarily of symptom management (45). There has since been extensive research and development of HEMT (46, 47). The newest combination (ETI) has been shown to safely improved lung function and sweat chloride concentration (48). Since its approval by the FDA in 2019, mortality and life expectancy has continued to improve (45). However, due to the nature of the novelty of these new treatments, not much is known at this time about the long-term adverse effects. Clinical trials have shown concern for “mental fogginess,” headache, and changes in mood (49, 50). Other studies have shown concern for weight gain, changes in bone density, and decreased exercise tolerance (51). In addition, ETI can affect liver function and can interact with numerous medications such as antifungals and antivirals and therefore, it is of utmost importance to either closely work with pharmacists or screen for all potential medication interactions. Overall, long-term effects of ETI are still largely unknown and will be of interest for future studies especially questions regarding pharmacokinetics since several pwCF are on reduced dosages. This will require even more attention in a CF aging population, since polypharmacy has been shown repeatedly to be a major concern for the elderly due the burden of taking multiple medications and their associated side effects, increased health care costs as well as reduced functional capacity and increased risk for delirium and falls (52, 53).

## CF and mental health/cognitive dysfunction

In adolescents with CF, it is reported that 10.2% have anxiety and 13.4% experience depression (4). As patients age, the rates only increase, with the percentage of adults with CF who reported depression and anxiety in 2021 was 29.6 and 28.0%, respectively (4). These rates are similar to other adults in the United States, reporting symptoms of depression or anxiety rates 24.5 to 36.5% depending on month surveyed (54). Notably in the CF population, it has been shown that clinically significant depressive symptoms are related to loss of lung function over time (55, 56). On the other hand, reported positive mental health and well-being has been associated with improved pulmonary function in pwCF (57). This association highlights the increased risk of depression and anxiety in pwCF. Additionally, although HEMT has shown symptomatic improvement in pwCF, there have also been reports of worsening anxiety and depression after starting these medications (58). Although this association is not yet well understood, it raises a need for more attention and follow up.

Furthermore, multiple studies have evaluated the relationship between eating disorders (ED) and disordered eating behaviors, with overall mixed results of either an increased or decreased rate of eating disorders in pwCF compared to control (59). Even in the absence of a strong association between ED and pwCF, in general, young people with chronic illnesses that require dietary adjustment or management may be at higher risk of disordered eating practices and eating disorders with increased rates of disturbances in eating behavior and in attitude have been seen in pwCF (60, 61). Knowing that there is a relationship between negative mental health and body image in pwCF is important, especially as more pwCF are treated with HEMT, which may only further exacerbate mood disorders while also increasing body weight.

Currently, guidelines recommend depression screening for all individuals 12 years and older with CF (62). Understanding the prevalence of depression and anxiety in adults with CF and the relationship between pulmonary function, highlights the importance of this screening (56). Integrative based care plans to provide multidisciplinary support have been shown to reduce both depressive symptoms as well as CF related symptoms (63). This will become even more important in an aging CF population, since neurodegenerative diseases will increase. Interestingly, CFTR is widely expressed in the nervous system, which itself exerts a widespread control of multiple organs and tissues (64) and Roy et al. have shown that pwCF show regional brain changes in sites that regulate cognitive, autonomic, mood and respiratory functions (65). Cognitive dysfunction has been also demonstrated in children with CF, which was associated with early malnutrition (66). For the future with pwCF on HEMT growing older, this will be of great importance to further delineate, since they are vulnerable for aging-related cognitive dysfunction, which will be affected by both HEMT and nutrition and chronic disease.

## Obesity

Historically, patients with CF have struggled with growth and nutritional status, with 16.2% of patients being classified as underweight (BMI < 18.5) in 2001 (4). With improvements in nutrition and dietary interventions such as individualized nutrition plans and high fat high carbohydrate diets, the percentage of people with underweight BMI has decreased to 4.1% in 2021 (4, 67). The CF Foundation has significantly contributed to those improvements with provision of guidelines, multidisciplinary CF clinics including dietitians and screening for food insecurities. Average BMI has increased from 21.3 to 23.4 in young adults (20–40 years) with CF over the past 15 years (4). In children aged 2–19 years, the median BMI percentile has increased from 47.7 in 2006 to 62.1 in 2021 (4). Additionally, 40.4% of adults have a BMI categorized as overweight (28.7%) or obese (11.7%), this may be associated to CFTR modulator therapy as HEMT therapy has shown to increase BMI (4, 7).

Additionally, when pwCF are started on HEMT therapy they tend to have increased forced expiratory ventilation in 1 s (FEV_1_) and there is evidence that they have an increased exercise capacity (7, 68). This relationship is important in considering other factors that may impact pwCF ability to maintain a healthy weight. Limitation of physical activity due to respiratory symptom burden has been hypothesized to result in decreased physical activity in CF patients compared to the general population (69, 70). Additionally, compared to healthy subjects, CF patients develop more hypoxia and hypercapnia during exercise, which may contribute to an decreased interest in physical activity (71). As the CF population ages, an important consideration is how HEMT may affect physical activity and how this will in turn affect BMI and rates of obesity in pwCF. As speculated above, since obesity rates will increase, especially in pwCF on ETI early on and in the developed countries, lipid disorders, the metabolic syndrome and diabetes incidences will be on the rise, which will increase the risk for cardiovascular disease and therefore will require special focus in the future.

## CF related bone disease (CFBD) and CF arthropathy (CFA)

CFBD and CFA are two different entities but are two major possible co-morbidities for pwCF. Similar to osteoporosis, CFBD is defined as low bone mineral density on dual energy x-ray absorptiometry (DXA); according to one study, roughly 25–30% of people experience bone disease (72). Current recommendations per the American CF guidelines recommend the screening for CFBD in pwCF using DXA starting at the age of 18 years of age (or 10 years earlier if at risk) and repeating every 1–5 years pending findings (73). Regarding treatment, bisphosphonates have been well studied with proven efficacy in the treatment of mineral bone disease in pwCF (74); however, there is limited data on the long-term use of bisphosphonates, raising concerns about unique complications in this population, especially now getting older and new strategies will need to be developed and personalized.

## Discussion

**Figure 1:**
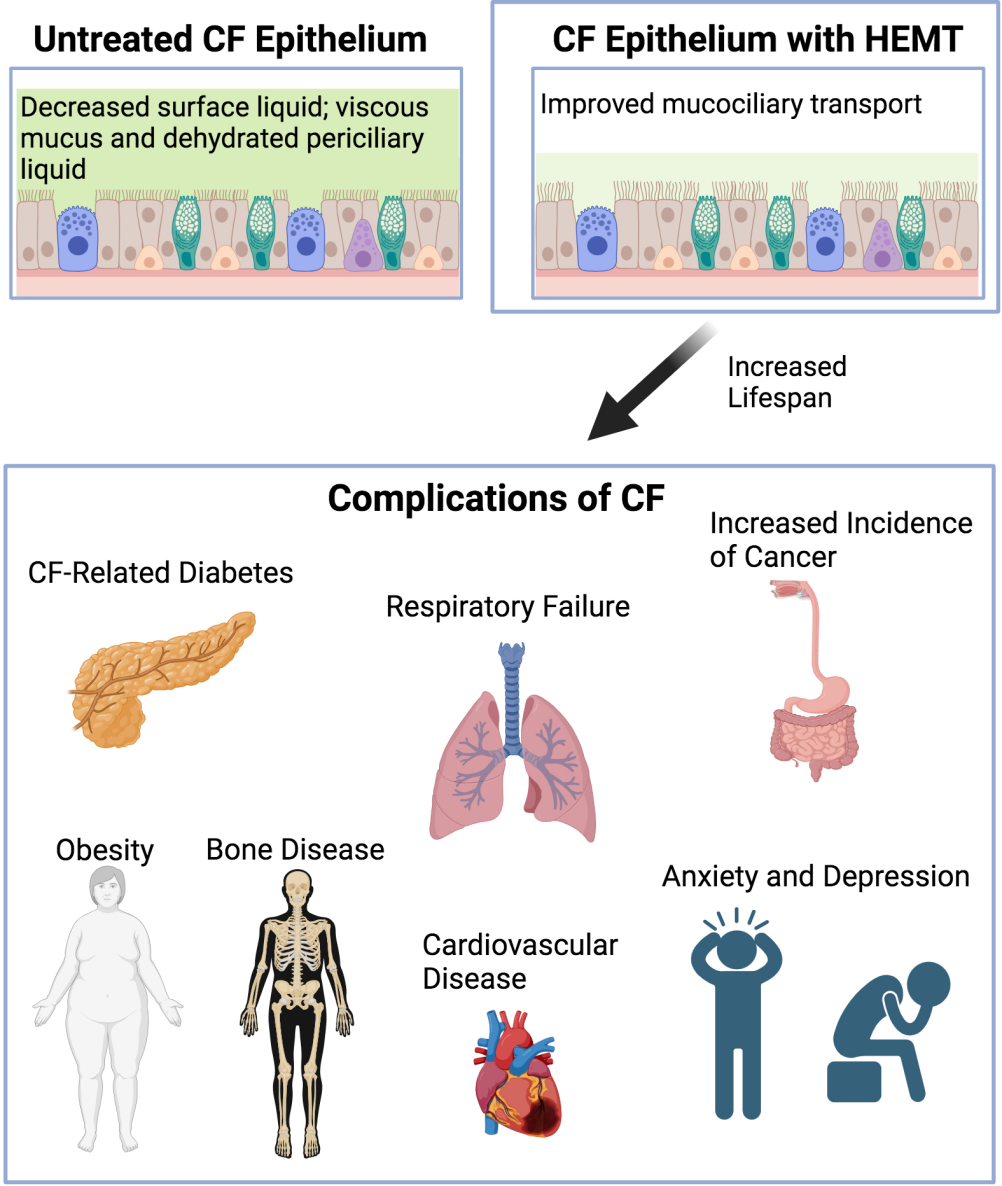
Diagram demonstrating graphically CF epithelium before and after HEMT and complications of CF arising with increased life span. Created with Biorender.com.

Overall, the development of CFTR modulator therapies have changed the landscape of the field and resulted in increased life expectancy for pwCF. Along with new developments come new challenges such as medication adverse effects plus learning how to address and treat common conditions of aging pwCF will now face. Known comorbidities and complications associated with CF such as CFRD, chronic lung disease, osteoporosis and polypharmacy will not completely go away and pathology and treatment options will potentially change or need to be adjusted in an aging CF population. Furthermore, new comorbidities that have not been that prevalent before, will emerge with age such as cognitive dysfunction, cardiovascular disease and obesity related complications (Figure 1). At this point, there is still much to learn. Given that in the past pediatricians have largely cared for these patients, internists, adult pulmonologists, and even geriatricians will soon be challenged to take over care of these patients as they grow older. Longitudinal studies are needed to follow along with these patients and identify how best to aid in these new challenges and enable pwCF to age in a healthy way.

## Author contributions

SB: Conceptualization, Writing – original draft, Writing – review & editing. AL: Writing – original draft, Writing – review & editing. EH: Conceptualization, Writing – original draft, Writing – review & editing. JP: Writing – review & editing, Visualization. BG: Writing – review & editing. GS: Writing – review & editing. SK: Writing – review & editing, Conceptualization, Funding acquisition, Writing – original draft.
